# The Sanatorium Treatment of Consumption


**Published:** 1904-02-27

**Authors:** T. N. Kelynack

**Affiliations:** Physician to the Mount Vernon Hospital for Consumption and Diseases of the Chest, Hampstead and Northwood, London.


### HOSPITAL CLINICS AND MEDICAL PROGRESS.

### THE SANATORIUM TREATMENT OF CONSUMPTION.*

By T. N. KELYNACK, M.D., M.R.C.P., Physician to the Mount Vernon Hospital for Consumption and Diseases of the Chest, Hampstead and Northwood, London.

LADIES AND GENTLEMEN,—During the present course of lectures various pathological and clinical aspects of pulmonary tuberculosis have been presented to you. In our out-patient department and at the hospital you have abundant opportunities open to you of examining the subjects of all forms of pulmonary consumption and presenting every manifestation of the disease, and you can watch the progress of the warfare, the issues of which are life and death.

I propose therefore to limit myself to a consideration of the open-air, hygienic or institutional treatment of consumption as now carried out in the sanatoria of this country.[1]

I have recently had opportunities of visiting the greater number of these establishments in Great Britain and Ireland and thoroughly investigating not only the structural features of these institutions, but seeing something of the methods of management, and learning much concerning the views and practice of those in charge. I thus venture to present to you in a necessarily condensed and I fear very incomplete form certain impressions that I have formed and some conclusions at which I have arrived.

I trust my brief survey and attempt at something of a forecast may at least suggest lines of investigation or stimulate inquiry among those, of whom I see a number present, who are responsible for the conduct of sanatoria or deeply interested in the adoption of natural measures and rational procedures aiming at the arrest of consumption and the practical restoration of the consumptive.

### THE EVOLUTION OF THE SANATORIUM.

While it is true that we owe much to our German *confrères* in the development of the institutional treatment of phthisis, and should on no account omit to render homage to such practical pioneers as Brehmer of Görbersdorf, Dettweiler of Falkenstein, and Walther of Nordrach, in all fairness we have no right to forget the far-seeing observers and clear-minded, independent, seer-like teachers of our own kin. British physicians and pathologists, especially when imbued with something of the prophet's spirit, are wont to receive but scant honour in their own land. But Englishmen should ever remember that one of their own countrymen, George Bodington,[2] mere village doctor though he may have been, was the first to seriously practise the treatment of phthisis by what he spoke of and which we now acknowledge, as "the natural method."

While we do well ever to strive after a full and complete scientific direction for our treatment we cannot close our eyes to the fact that much success may accrue even when our pathological knowledge is imperfect and sometimes even when our views are actually erroneous. Such I think it must be admitted was the case with Bodington. In the introduction to his now classic monograph,[3] in which he so trenchantly attacked the hopeless inactivity and apathetic indifference then prevalent, he indicated his theoretical point of view:—

"It will be observed," he writes, "that the main ground of the treatment has been to preserve or restore to a normal condition the functions of the nervous filaments interwoven with the substance of the lungs, and exercising influence over the capillary system and other parts of the organisation. It has been assumed that the first link in the chain of morbid actions arises there, as they first feel the irritation from the presence of the morbid matter deposited as a foreign body, and that all the other changes are consecutive to this wasting or distribution of the nervous energy of the filaments with which the tuberculous matter comes in contact. Upon this view the treatment of pulmonary consumption in the way herein recommended has been founded."

But Bodington, nevertheless, seized the essential elements and used them with much success, as his clinical record of cases fully attests. Permit me to make but a few quotations which will be sufficient evidence to indicate in what a remarkable manner our modern hygienic methods of procedure have been to a great extent anticipated by George Bodington:—

"To live in and breathe freely the open air, without being deterred by the wind or weather, is one important and essential remedy in arresting its progress."

"The cold is never too severe for the consumptive patient in this climate; the cooler the air which passes into the lungs the greater will be the benefit the patient will derive."

"The common hospital in a large town is the most unfit place imaginable for consumptive patients, and the treatment generally employed there very inefficient, arising from the inadequacy of the means at command."

"I think in the neighbourhood of every large town, sufficiently distant to be clear of its contamination from smoke, etc., and in well-chosen spots, medical men should be established with all the means about them for the treatment of the disease in question, to whom those who live in the towns should confide their patients of this kind, at the same time rendering them the benefit of their advice as far as needful, rather than that they should be dismissed to the care of nurses and lodging-house keepers in distant

* A Lecture delivered at the Central Out-patient Department of the Mount Vernon Hospital, 7 Fitzroy Square, W., on Tuesday, November 26th, 1903, in connection with the Winter Course of Post-Graduate Lectures.

[1] A fairly complete list of sanatoria for consumptives in this country will be found in the *Medical Annual* for 1904. I have published particulars concerning a considerable number of these institutions in the *Medical Press and Circular*, July 1st, 1903, *et seq.* Useful details may also be found in Dr. F. Rufenacht Walters' work "On Sanatoria for Consumption," second edition, Lond. 1901; and in *The Sanatoria Annual*, 1902.

[2] A short biographical notice of George Bodington appeared in the *British Medical Journal* of March 11th, 1882.

[3] "The Treatment and Cure of Pulmonary Consumption," 1840. For readily accessible reprint see "Selected Essays and Monographs chiefly from English Sources." London: New Sydenham Society, 1901, p. 125.

situations; and again I repeat I do think that for the poorer classes, on account of the magnitude of the evil as regards them, hospitals especially for their use and treatment ought to be established in fit situations."

Much also in the mental conceptions of Henry MacCormac[4] of Belfast, regarding the pathology of phthisis was bizarre and we now believe erroneous and yet he laboured indefatigably with his pen, quaint and erratic though the product may often have been, to secure a recognition of the value of hygienic means in the arrest of consumption.

The admirable pioneer work of the late Sir Benjamin Ward Richardson in regard to open-air treatment I have been surprised to find is but little known even by those engaged in the conduct of sanatoria and yet his monograph,[5] published in 1857, breathes the spirit and sentiment of to day.

Let the following extracts stand as evidence:—

"In a cosy room the consumptive is bound never to live, nor in any room, indeed, for great lengths of time. So long as he is able to be out of doors, he is in his best and safest home."

"Stoves of all kinds, heated pipes, and in a word, all modes of supplying artificial warmth, except that by the radiation from an open fire, are, according to the facts which I have been able to collect, injurious."

"If special hospitals for consumptives are to be had, they should be as little colonies, situated far away from the thickly populated abodes of men, and so arranged that each patient should have a distinct dwelling-place for himself. They should be provided with pleasure-grounds of great extent, in which the patients who could walk about should pass every possible hour in the day; and with glass-covered walks overhead, where the open air could be freely breathed, even if rain were falling."

Richardson also in his remarkable and almost prophetic essay shows how before his day and even before the time of Bodington eager minds had travelled far towards accepting a rational view of managing consumptives.

Parrish,[6] writing in 1830, stated his opinion thus:—

"Vigorous exercises, and a free exposure to air, are by far the most efficient remedies in pulmonary consumption. It is not, however, that kind of exercise usually prescribed for invalids—an occasional walk or ride in pleasant weather, with strict confinement in the intervals—from which much good is to be expected. Daily and long-continued riding on horseback or in a carriage is, perhaps, the best mode of exercise; but where this cannot be commanded, unremitting exertion of almost any kind in the open air, amounting even to labour, will be found highly beneficial. Nor should the weather be scrupulously studied. Though I would not advise a consumptive patient to expose himself recklessly to the several inclemencies of the weather, I would, nevertheless, warn him against allowing the dread of taking cold to confine him on every occasion when the temperature may be low, or the skies overcast."

"I may be told that the patient is often too feeble to be able to bear exertion; but except in the last stage, where every remedy must prove unavailing, I believe there are few who cannot use exercise out of doors; and it sometimes happens that those who are exceedingly debilitated find, upon making the trial, that their strength is increased by the effort, and that the more they exert themselves the better able they are to support the exertion."

### The Modern Attitude to Sanatoria.

But attractive and valuable though a study of the history of the hygienic treatment of consumption may be, it is not so much with the mental conception of the past that we are concerned as with the practical methods of the present and the tendency of thought and action in the future.

Much has been published on almost every phase of sanatorium treatment. Numerous important congresses[7] have been held, and an extensive literature has rapidly come into being.[8]

Many excellent periodicals[9] are more or less devoted to the subject, and in the medical and lay press the question of the best means of dealing with consumption is receiving prominent attention.

And yet in spite of all we are still in much uncertainty as to our theoretical beliefs, and our practice is conspicuous by a lack of any really scientific uniformity.

Although the general principles of the hygienic treatment of consumption are often spoken of as though one might consider them as fairly established, it cannot be denied that much in its actual conduct and especially in the application of details to individual cases, we are still to a very great extent only in the experimental stage. The records of a symposium on the present treatment of consumption held recently before the Hunterian Society may well be consulted.[10]

The publication of the prize essays,[11] sent in competition, in connection with the establishment of King Edward VII. Sanatorium, and numerous other articles,[12] dealing with sanatorium construction and management have gone far to stimulate inquiry and arouse serious study regarding both the principles and details of "the natural method" of dealing with consumption.

Much further advance may be expected in the near future. The report of the Royal Commission

[4] "Consumption, as Engendered by Rebreathed Air." By Henry MacCormac, M.D. London: Longmans, 1855.

[5] "The Hygienic Treatment of Pulmonary Consumption." By Benjamin W. Richardson, M.D. London: John Churchill, 1857. See also *Medical Press and Circular*, June 10th, 1903.

[6] *North American Medical and Surgical Journal*, 1830. Quoted by B. W. Richardson in his "Hygienic Treatment of Pulmonary Consumption," London: 1857.

[7] "Transactions of the British Congress on Tuberculosis," London, 1902.

[8] Consult bibliography given in Dr. Latham's and Mr. West's "Prize Essay on the Erection of the King's Sanatorium for Consumption," London, 1903. See also "Tuberculosis," by Norman Bridge, A.M., M.D., Philadelphia, 1903; "La Cure de la Tuberculose dans les Sanatoriums Français," by A. F. Plicque and Verhaeren, Paris, 1903; "The Principles of 'Open-air' Treatment of Phthisis and of Sanatorium Construction," by Arthur Ransome, M.D., F.R.S. London, 1903 and "The Prevention of Consumption." By Alfred Hillier, M.D. London: 1903. "La Cura del Tubercolotico Pulmonare nel Sanatorio," by Prof. Vincenzo Cozzolino. Torino: 1901.

[9] *Zeit f. Tuberculose, und Heilstättenwesen*; *Tuberculosis*, the monthly publication of the Central International Bureau for the Prevention of Consumption, Leipszig, J. A. Bartle; *Revue de Tuberculose*; and the *Journal of Tuberculosis.*

[10] *Transactions Hunterian Society*, London: 1903.

[11] *Lancet*, London: January 3rd, 1903.

[12] See article by T. G. Lyon, M.D., *Tuberculosis*, January, 1903; "A Model Sanatorium for Tuberculosis," by A. G. R. Foulerton, F.R.C.S., and R. Langton Cole, F.R.I.B.A., *Public Health*, March, 1903.

on Tuberculosis is eagerly awaited. Meanwhile many important contributions to the pathology of tuberculosis are being made, many of which, however, it must be admitted, tend rather to disturb the crystallization of thought into forms which may be of service in defining sure paths along which effective advance may proceed.

At the present time it is difficult to make a forecast as to the future of sanatorium treatment in this country. Much uncertainty still remains as to our knowledge respecting the nature of the seed, the character of the soil, and the extent of the harvest. While Koch still holds that the chief danger of infection is from the sputum and from person to person, Von Behring[13] appears to contend that all forms of tuberculosis depend on an infection by way of the intestinal tract contracted early in life although often long remaining latent, and H. C. Bastian[14] would seem to hold to the almost inconceivable view of an origin *de novo.*

The ultimate effect of these discordant views on actual practice it is difficult to estimate.

Manifestly much remains to be revealed as to conditions of life and variations in vitality which modify the natural resistance of the soil as well as the various circumstances which lessen the virulency of the seed before hygienic treatment can be said to have escaped from the trammels of empiricism. But although it is very necessary constantly to remind optimists that in open-air treatment there is nothing which must be considered as a specific for the cure of consumption, and while we must admit that knowledge of its ætiology and precise information concerning its pathology is by no means complete, nevertheless the manifest results of institutional treatment would seem to afford substantial evidence for the opinion that in a rationally conducted course of hygienic treatment we have the most reliable means of securing an arrest of pulmonary tuberculosis.

13 *Deutsche Medicinische Wochenschrift*, September 24th. 1903.

14 *Lancet*, October 31st, 1903.

*(To be continued.)*

